# Improving the Identification and Coverage of Plant Transmembrane Proteins in *Medicago* Using Bottom–Up Proteomics

**DOI:** 10.3389/fpls.2020.595726

**Published:** 2020-12-18

**Authors:** Han Chung Lee, Adam Carroll, Ben Crossett, Angela Connolly, Amani Batarseh, Michael A. Djordjevic

**Affiliations:** ^1^Division of Plant Sciences, Research School of Biology, College of Medicine, Biology and the Environment, The Australian National University, Canberra, ACT, Australia; ^2^ANU Joint Mass Spectrometry Facility, Research School of Chemistry, College of Science, The Australian National University, Canberra, ACT, Australia; ^3^Sydney Mass Spectrometry, The University of Sydney, Sydney, NSW, Australia; ^4^BCAL Diagnostics, National Innovation Centre, Eveleigh, NSW, Australia

**Keywords:** transmembrane protein, transmembrane domain, liquid chromatography, mass spectrometry, detergent-free purification, *Medicago truncatula*, TRAMDOMI algorithm

## Abstract

Plant transmembrane proteins (TMPs) are essential for normal cellular homeostasis, nutrient exchange, and responses to environmental cues. Commonly used bottom–up proteomic approaches fail to identify a broad coverage of peptide fragments derived from TMPs. Here, we used mass spectrometry (MS) to compare the effectiveness of two solubilization and protein cleavage methods to identify shoot-derived TMPs from the legume *Medicago.* We compared a urea solubilization, trypsin Lys-C (UR-TLC) cleavage method to a formic acid solubilization, cyanogen bromide and trypsin Lys-C (FA-CTLC) cleavage method. We assessed the effectiveness of these methods by (i) comparing total protein identifications, (ii) determining how many TMPs were identified, and (iii) defining how many peptides incorporate all, or part, of transmembrane domains (TMD) sequences. The results show that the FA-CTLC method identified nine-fold more TMDs, and enriched more hydrophobic TMPs than the UR-TLC method. FA-CTLC identified more TMPs, particularly transporters, whereas UR-TLC preferentially identified TMPs with one TMD, particularly signaling proteins. The results suggest that combining plant membrane purification techniques with both the FA-CTLC and UR-TLC methods will achieve a more complete identification and coverage of TMPs.

## Introduction

Transmembrane proteins (TMPs) play critical roles in the function of all living organisms. Approximately 20 to 30% of the sequenced genomes from microbial or eukaryotic organisms encode TMPs (Komatsu et al., 2007; Schey et al., 2013; Almeida et al., 2017). In humans, roughly 50% of all known drug targets are TMPs (Hopkins and Groom, 2002; Lappano and Maggiolini, 2011; Tautermann, 2014), and many human diseases are caused by malfunctioning TMPs (Hardy, 2017; Hasegawa et al., 2017; Hattori et al., 2017; Szablewski, 2017). In plants, TMPs play important roles in solute transport (Pellizzaro et al., 2015), signal recognition and transduction (Kemmerling et al., 2011; Mohd-Radzman et al., 2015; Song et al., 2015; Imin et al., 2018), growth and development (Clark et al., 1997; van der Knaap et al., 1999; Osakabe et al., 2005; ten Hove et al., 2011), and photosynthesis (Liu and Last, 2015; Okumura et al., 2016).

TMPs can be difficult to identify by proteomic strategies due to their high hydrophobicity (Seddon et al., 2004; Carpenter et al., 2008; Rawlings, 2016) and low abundance (Vit and Petrak, 2017). All proteomic strategies aimed at identifying TMPs begin with membrane enrichment that involves the purification of microsomal, organelle, or plasma membranes (Mirza et al., 2007; Ogawa et al., 2008; Huang et al., 2013; Abdallah et al., 2014; Guillier et al., 2014; Avila et al., 2015; Aloui et al., 2018). The effectiveness of the proteomic identification of TMPs from these membrane-enriched fractions, however, is compromised by contaminating cytoplasmic or membrane-associated proteins without transmembrane domains (TMDs). High ionic strength buffers can remove some of these contaminants with varied degrees of success (Marx et al., 2016; Vit and Petrak, 2017). Typically, TMPs represent roughly 20% of proteomic identifications (Chen et al., 2008). Although trypsin’s high cleavage specificity and efficiency makes it the gold standard enzymatic method for MS-based bottom up proteomics (Olsen et al., 2004), its use typically enables sequence coverage limited to the soluble loops and terminal tails of TMPs (Schey et al., 2013). In addition, TMPs are difficult to solubilize and digest using standard urea solubilization and trypsin Lys-C-based procedures. Various detergents, chaotropic agents, organic solvents as well as proteinases and chemical cleavage reagents have been used to help solubilize and/or digest TMPs (Bennett et al., 1992; Newby et al., 2009; Guillier et al., 2014). There are few reports, however, comparing the efficiency of urea and acid-based procedures that aim to identify plant TMPs using mass spectrometry (MS).

To determine the protein makeup of a given sample using bottom–up proteomics requires maximal peptide coverage of the sample. TMPs with a high content of TMDs are underrepresented in MS identification since the most used protein purification method for bottom–up proteomics uses 8 M urea to solubilize the sample. The poor solubilization and thus poor trypsin digestion (UR-TLC) reduces the identification rate for proteins with a large number of TMPs (Long et al., 2018). The inability of urea to dissolve the membrane most likely contributes to trypsin Lys-C failing to cleave sites including TMDs. To address the poor ability of 8 M urea to solubilize hydrophobic TMPs, many proteomic studies use certain kinds of detergents to provide solubility (Kar et al., 2017). It is well accepted, however, that different detergents selectively solubilize certain proteins and not others (Churchward et al., 2005; Chen et al., 2007; Arachea et al., 2012; Laganowsky et al., 2013; Tanca et al., 2013). For example, hydrophobic membrane proteins in lipid rafts are poorly solubilized by detergents (Morel et al., 2006; Casem, 2016; Kusumi et al., 2020). In addition, detergents are not MS friendly and must be removed prior to MS analysis (Zhang and Li, 2004; Yeung et al., 2008). A previous *Medicago truncatula* study utilized an 8 M urea-based approach to solubilize and identify a wide range of proteins from different tissues (Marx et al., 2016). Here, we established a non-detergent proteomic approach by using FA to substitute for detergents and 8 M urea (Zhao et al., 2013) for the solubilization of membrane samples of Medicago.

To address the shortcomings of urea and detergent-based approaches, we designed a non-detergent-based purification and identification strategy to analyze TMPs in Medicago using MS. *Medicago* is an important nitrogen-fixing agricultural crop, and this study augments the prior proteomic analyses of this plant (Natera et al., 2000; Djordjevic et al., 2003, 2007; Djordjevic, 2004; Zhang et al., 2006; Kusumawati et al., 2008; Lee et al., 2013; Marx et al., 2016). We used microsomal membrane preparations from *Medicago* shoot tissue as a common starting material. All the proteins from microsomal membrane preparation were precipitated by trichloroacetic acid (TCA). The protein samples were divided and then subjected to (i) the popular urea solubilization and trypsin Lys-C digestion-based method or (ii) a method for improving the cleavage of TMPs, which utilizes formic acid (FA) solubilization followed by cyanogen bromide (CNBr) cleavage and then trypsin Lys-C digestion (Quach et al., 2003; Girolamo et al., 2010; Vit and Petrak, 2017). It is well recognized that urea solubilization followed by enzymatic cleavage has a low efficiency in solubilizing and cleaving TMPs (Seddon et al., 2004; Carpenter et al., 2008; Rawlings, 2016). The cleaved peptides derived from the two methods were separated by high-pH reversed-phase peptide fractionation, and identified by orbitrap-based MS. The effectiveness of the two methods was assessed by (i) comparing total protein identifications, (ii) determining how many TMPs were identified, and (iii) defining how many peptides incorporate all, or part, of the TMD sequences using a new algorithm. In addition, we determined the subcellular location and predicted the function of the proteins identified. The objective of this study was to establish a detergent-free and effective strategy to identify plant TMPs and improve the overall identification and coverage of these proteins using MS.

## Materials and Methods

### Plant Growth

Surface-sterilized *M. truncatula* cv. Jemalong A17 seeds (Imin et al., 2013) were germinated and grown on Fåhraeus medium plates (Mohd-Radzman et al., 2015). Eight seedlings per plate were grown for 14 days in a Conviron growth chamber at 25°C with a 16-h photoperiod and a photon flux density of 100 μm mol m^–2^ s^–1^ (Imin et al., 2013). Shoots were harvested separately and frozen in liquid nitrogen for immediate extraction or stored at −80°C before use. Three independent batches of Medicago shoot samples were collected to achieve independent biological replicates and enable an assessment of significance.

### Microsomal Membrane Preparation

Proteins were extracted from homogenized and ground tissue based on published methods (Marx et al., 2016) with slight modifications. In brief, in order to have approximately 100 mg of microsomal membrane (MM) material, 12 g of shoot tissues (leaves and cotyledons) from 14-day-old seedlings were ground into a fine powder in liquid nitrogen using a mortar and pestle. After grinding, five volumes (*circa* 50 ml) of ice-cold extraction buffer [290 mM sucrose, 250 mM Tris (pH 7.6), 25 mM EDTA (pH 8.0), 10 mM KCl, 25 mM NaF, 50 mM sodium pyrophosphate, 1 mM ammonium molybdate, 1 mM PMSF, mini EDTA-free protease inhibitor (Roche)] was added to the ground plant tissue samples. The ground tissue was further homogenized by repeated probe sonication (MSE: Imgen technologies) (10 cycles of 1 min sonication on ice followed by a 30-s rest period on ice). The homogenized plant tissue was filtered through a 100-μm filter (BD Falcon, Bedford, MA, United States) and subsequently centrifuged for 10 min (4,000 *g*, 4°C) to remove the remaining tissue debris. MMs were prepared by ultracentrifugation for 30 min at 100,000 *g* (4°C) to remove cytoplasmic proteins. After ultracentrifugation, the MM pellet was resuspended in 1 M Na_2_CO_3_ (pH 11) and incubated on ice for 5 min to remove weakly associated proteins (Huang et al., 2013). After incubation, the MMs were subject to ultracentrifugation (100,000 g, 4°C) for 30 min (Abas and Luschnig, 2010).

### TCA Precipitation and Protein Solubilization

*Medicago* MM proteins were purified by TCA precipitation (Link and LaBaer, 2011) with a slight modification to remove the non-protein contamination. In brief, 500 μl of 11% TCA was added into MM sample pellet and incubated on ice for 20 min. Another 500 μl of ice-cold 10% TCA solution was added, and the sample was incubated at −20°C overnight. The solution was centrifuged at 20,000 *g* for 30 min to recover the precipitated protein and the supernatant discarded. The protein pellet was rinsed three times with 80% acetone (Marx et al., 2016), centrifuged at 20,000 *g* for 10 min and dried using a vacuum evaporator (VirTis, bench TopK). The protein sample was divided into two to assess the effectiveness of the two protein solubilization and cleavage protocols.

### Urea Solubilization Followed by Trypsin -Lys-C Digestion (UR-TLC)

The dried protein pellet was re-solubilized in 1 ml of dissolving buffer: 8 M urea, 50 mM Tris–HCl (pH 8.0), 30 mM NaCl, 1 mM CaCl_2_, 20 mM sodium butyrate, 10 mM nicotinamide, mini EDTA-free protease inhibitor (Roche). To improve protein solubility, protein samples were subjected to repeated probe sonication (10 times for 10 s of pulse and 10 s of rest on ice). Protein concentration was estimated using a Bradford assay (Bio-Rad). Proteins were reduced with 5 mM dithiothreitol at 60°C for 40 min. The reduced proteins were alkylated with 15 mM iodoacetamide in the dark at room temperature for 40 min. Alkylation was quenched by adding 5 mM dithiothreitol and incubated at room temperature for 15 min. The protein solution concentration was estimated by UV 280 absorbance, and 200 μg of protein sample was enzymatically digested in a two-step process using a Trypsin-Lys-C mix (Promega). A 25:1 molar ratio of the enzyme was added to the protein solution and digested for 3 h at 37°C. After 3 h, the urea concentration was adjusted to 2 M by dilution with 50 mM Tris, pH 8.0 and the reaction kept at 37°C overnight. After overnight digestion, the sample was run over a Sep-Pak C18 classic cartridge (Waters, Milford, MA, United States) to remove the salts, and the peptides were eluted using 100% acetonitrile (ACN).

### Formic Acid Solubilization Followed by Initial CNBr Cleavage and Trypsin-Lys-C Digestion (FA-CTLC)

The dried protein pellet was dissolved in 500 μl of 70% FA using probe sonication and chemically digested with CNBr (Sigma) with a 100-fold molar ratio excess to the amount of starting dried protein (Wong and King, 2015). The CNBr solution was prepared as described (Washburn et al., 2001; Crimmins et al., 2005). Essentially, CNBr crystal was dissolved in ACN to make a 5 M final concentration. After 24 h of incubation at room temperature in the dark, the supernatant was collected by centrifugation at 20,000 *g* for 10 min, and the FA and CNBr were safely removed by lyophilization using cold trap. The dried peptide was dissolved in 10% ACN and 25 mM ammonium bicarbonate. The sample was reduced, alkylated, and then digested with Trypsin Lys-C, before being lyophilized (see above). The effectiveness of each procedure was assessed by centrifuging (10 min, 10,000 *g*) after UR-TLC or FA-CTLC treatments and examining the residual pellet. The residual pellet that remained after the UR-TLC was subjected to a further FA-CTLC procedure to validate that the pellet contained poorly solubilized undigested protein. The remaining CNBr solution was destroyed by adding 5 volumes of 1 M sodium hydroxide (Lunn and Sansone, 1985) before being disposed into chemical waste containers.

### Reverse-Phase High-pH Fractionation

The peptide digests were separated into eight fractions using the high-pH reversed-phase peptide fractionation Kit (Thermo Fisher Scientific, Waltham, MA, United States) (Kulak et al., 2017; Baldan-Martin et al., 2018). In brief, the C-18 spin column was equilibrated with 0.1% trifluoroacetic acid (TFA) before the digested peptides were loaded. Peptide samples (100 μg) dissolved in 0.1% TFA were loaded onto the spin column and washed with MilliQ H_2_O. The peptides were eluted into 16 fractions of increasing concentrations of ACN in 0.1% Triethylamine: 5% ACN (fraction 1), 7.5% ACN (fraction 2), 10% ACN (fraction 3), 12.5% ACN (fraction 4), 15% ACN (fraction 5), 17.5% ACN (fraction 6), 20% ACN (fraction 7), 25% ACN (fraction 8), 30% ACN (fraction 9), 35% ACN (fraction 10), 40% ACN (fraction 11), 45% ACN (fraction 12), 50% ACN (fraction 13), 60% ACN (fraction 14), 70% ACN (fraction 15), and 95% ACN (faction 16). The 16 fractions were collected and then recombined into eight fractions. Final fraction 1 comprised RP fractions 1 and 16; final fraction 2 comprised RP fractions 2 and 15; and so on until final fraction 8 comprised RP fractions 8 and 9. The fractionated peptides were lyophilized to remove the solvent, re-dissolved into 100 μl of 0.1% TFA, and cleaned up by C18 Ziptip (5 μg loading capacity, Merck Millipore, Burlington, MA, United States), and 1.7 μg of the sample was subjected to MS analysis.

### Mass Spectrometry

The liquid chromatography (LC) was performed by using Thermo Scientific UltiMate^TM^ 3,000 RSLCnano system with the setting at 60°C with customized columns. The columns were packed in-house using a laser puller and a pressure bomb, and the length of the columns were generally 35–40 cm with a 75-μm ID fused silica housing. The packing material used was Reprosil-Pur 120 C18-AQ, 1.9-μm particle size. The digested peptides were initially loaded onto the LC system with the mobile phases as 95% buffer A (0.1% formic acid/water) and 5% buffer B (0.1% formic acid/80% ACN/water). Samples were reconstituted in 10 μl of loading buffer (as above) and 3 μl directly injected for each run. Peptides were eluted with a 5–40% buffer B gradient for 90 min. The total acquisition time was 140 min, including a 95% ACN wash and re-equilibration. The LC was coupled to a Q-Exactive Plus Orbitrap mass spectrometer (Thermo Fisher Scientific, Waltham, MA, United States). MS scans acquired in the Orbitrap (mass resolution was 70,000 at m/z 200; mass analyzer range was m/z 350–2,000). The 20 most intense ions with a charge state ≥1 were fragmented in the high-energy C-trap dissociation collision (HCD) cell, and subsequently, tandem mass spectra were acquired in the Orbitrap mass analyzer with a resolution of 35,000 at *m*/*z* = 200.

### Data Analysis

All raw files generated by LC-MS/MS were processed by Proteome Discoverer 2.1 (Thermo Fisher Scientific) using the Sequest HT data analysis program to search against the *Medicago* protein sequence databases (UniProt, 2014.12.18.) (Bairoch et al., 2005). Database searching against the corresponding reversed database was also performed to evaluate the false discovery rate of peptide identification. The search parameters of Sequest HT were set as follows: precursor ion mass tolerance ±10 ppm and product ion mass tolerance of 0.05 m/z units. The cleavage specificity was set up as Trypsin/LysC: C-terminal of arginine and lysine, and CNBR/Trypsin/LysC: C-terminal of methionine, arginine, and lysine. Standard peptide modification was as follows: carbamidomethylation (CAM) at cysteine residues was set as a fixed modification, while oxidation at methionine, lysine, and proline residues, *C*-terminal amidation and deamidation at asparagine and glutamine, as well as *N*-terminal glutamine to pyroglutamic acid were set as variable modifications. When CNBr was chosen as the cleavage agent, methionine was set to be homoserine (Met- > Hse) or homoserine lactone (Met- > Hsl) as a variable modification. The O-formylation at serine and threonine, which was caused by formic acid (Zheng and Doucette, 2016), was set to be a variable modification. The phosphorylation at serine, threonine, and tyrosine was also set to be variable modification. For all experiments, we used Peculator with a strict cutoff (<0.1) to determine the FDR of the peptides identified. Due to sequence redundancy, the proteins that had shared the same set of identified peptides were grouped into protein groups.

### TMD Prediction

The TMD prediction was done by using the TMHMM Server, v. 2.0^1^. TMHMM is a membrane protein topology prediction method based on a hidden Markov model (Sonnhammer et al., 1998). The web server-based search engine correctly predicts 97–98% of the transmembrane helices and can distinguish between soluble and membrane proteins with a specificity and sensitivity better than 99% (Krogh et al., 2001).

### TMD (TRAMDOMI, TRAns Membrane Domain Motif Identification)

The TMD analysis was annotated by a customized python script. In brief, two peptide sequence sets were prepared for TMD mapping. One was the detected peptide set, which was derived from the MS analysis of the samples. The second one was the complete *Medicago* TMD motif set, which was predicted by TMHMM server using the *M. truncatula* sequence databases (UniProt, 2014.12.18). TMD identification was done by mapping the detected peptide set to the *Medicago* TMD motif set. The mapping rules were defined as followed. Any detected peptide that satisfied one of the following rules was considered a hit:

1. The length of the detected peptide derived from MS encompassed the complete predicted *Medicago* TMD sequence or laid within the predicted TMD.
2. The detected peptide derived from MS extends from a position outside the TMD to within the TMD with a minimum of a two-amino acid overlap, or started within the TMD and extended to a position outside of the TMD with a minimum of two-TMD-amino acid overlap.

### Calculate the Grand Average of Hydropathy Value for Protein Sequences

The Grand Average of Hydropathy (GRAVY) value is calculated by the sum of hydropathy values of all amino acids divided by the protein length (Kyte and Doolittle, 1982). Hydrophobicity score (arbitrary unit) below 0 is more likely cytoplasmic protein (hydrophilic protein), while scores above 0 are more likely TMPs (hydrophobic) (Magdeldin et al., 2012).

### Subcellular Location Prediction

The subcellular protein location prediction was done by using LOCALIZER, a machine learning method for predicting subcellular protein localization in plant cells and is available at http://localizer.csiro.au/. It identifies proteins localized to chloroplasts and mitochondria by identifying the presence of transit peptides, and nucleus by using a collection of nuclear localization signals. It can achieve a prediction accuracy of over 90% for chloroplast and mitochondria, and 73% for nuclear proteins (Sperschneider et al., 2017). The queries of protein sequence were submitted directly to the server, and full plant sequences were chosen to perform the prediction.

### Protein Functional Annotation

The functional annotation was done using Mercator: http://mapman.gabipd.org/web/guest/app/Mercator. Mercator is a web based annotation application that achieves accuracies above 90% in predicted functional annotations when compared to manual annotation (Lohse et al., 2014). The queries of protein sequence were submitted directly to the server, searched against the database including TAIR Release 10 and SwissProt/UniProt plant proteins database, and classified into functional plant categories according to MapMan BINs (Thimm et al., 2004).

## Results

### Establishment of a FA-CTLC Method for Improving Bottom–Up Proteomics and Membrane Protein Identification

As a preliminary assessment step, we determined the effectiveness of FA solubilization combined with CNBr treatment to validate that CNBr cleaves to the *C*-terminal side of the comparatively rare methionine (Met) residues to generate large peptide fragments, since methionine occurs, on average, at every 50 amino acids. The MS results confirmed that FA solubilization followed by CNBr cleavage alone resulted in the production of large peptides with *C*-terminal Met residues, as expected (Supplementary Table 1). Peptides of large molecular mass with an uncharged, *C*-terminal Met do not ionize well and give poor-quality MS/MS spectra. As expected, this resulted in poor coverage of the proteome; only 2,566 protein groups were observed using the CNBr cleavage method only (at a 1% FDR). This was remedied by following the CNBr cleavage with trypsin Lys-C digestions.

### The FA-CTLC Is Superior at Solubilizing and Digesting Proteins From MM Preparations

**FIGURE 1:**
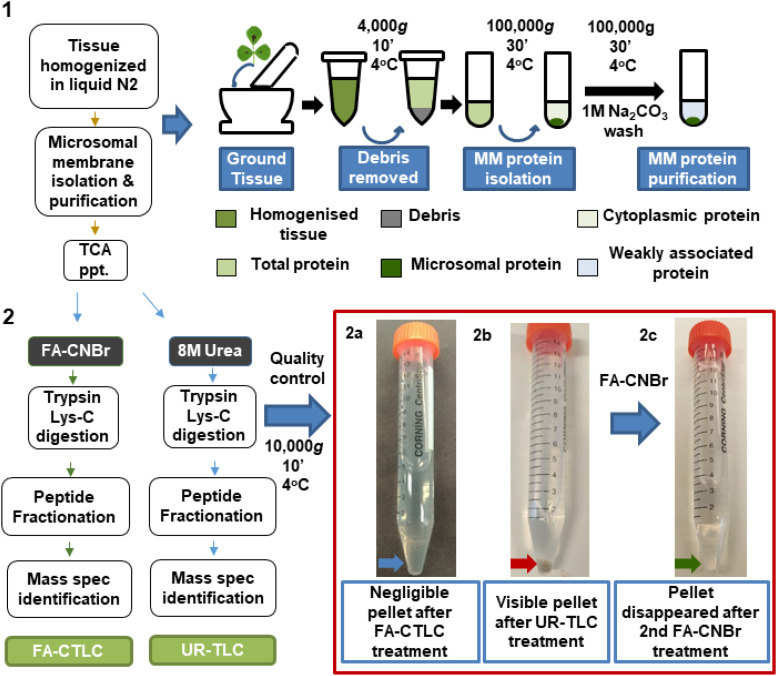
Flow chart summarizing the two solubilization and protein cleavage methodologies used in this study. Red box: Negligible insoluble material remained after using the formic acid solubilization, cyanogen bromide, and trypsin Lys-C (FA-CTLC) method (2a, blue arrow), whereas significant insoluble material remained after using the urea solidilization, trypsin Lys-C (UR-TLC) method (2b, red arrow). The remaining insoluble material after UR-TLC was solubilized and digested using the FA-CTLC method. Mass spectrometry (MS) analysis showed the presence of a wide range of proteins in the pellet (Supplementary Table 2). Subsequent centrifugation of this re-solubilized and FA-CTLC-treated material showed that negligible insoluble material remained (2c, green arrow).

A summary of the key steps of the two procedures is shown in Figure 1. To compare the effectiveness of the solubilization and digestion, MM preparations were split in half and subjected to either the UR-TLC or the FA-CTLC method. The results (Figure 1, red box; red arrow) showed that a considerable pellet of insoluble material remained after UR-TLC, but not after FA-CTLC. A second round of the UR-TLC was applied to the pellet, but after overnight digestion and subsequent re-centrifugation, the pellet remained. By contrast, the application of the FA-CTLC method to the insoluble pellet that remained after using the UR-TLC method resulted in no observable pellet after centrifugation (Figure 1, red box; green arrow). An MS analysis of this FA-CTLC re-solubilized pellet material (red box; red arrow) identified 5,644 protein groups (at a 1% FDR) in the insoluble material that remained after UR-TLC (Supplementary Table 2). From the 5,644 protein groups identified by a re-extraction of the insoluble material, the UR-TLC failed to identify 979 protein groups (Supplementary Table 3). This demonstrated that considerable protein material remained in the UR-TLC pellet (red box; red arrow) and that the FA-CTLC is more effective in solubilizing and digesting the proteins from MM preparations as shown in the method flowchart (Figure 1).

### Assessment of Inter-Sample Reproducibility and the Effectiveness of Protein Identification by MS After Using the FA-CTLC or the UR-TLC Method

**FIGURE 2:**
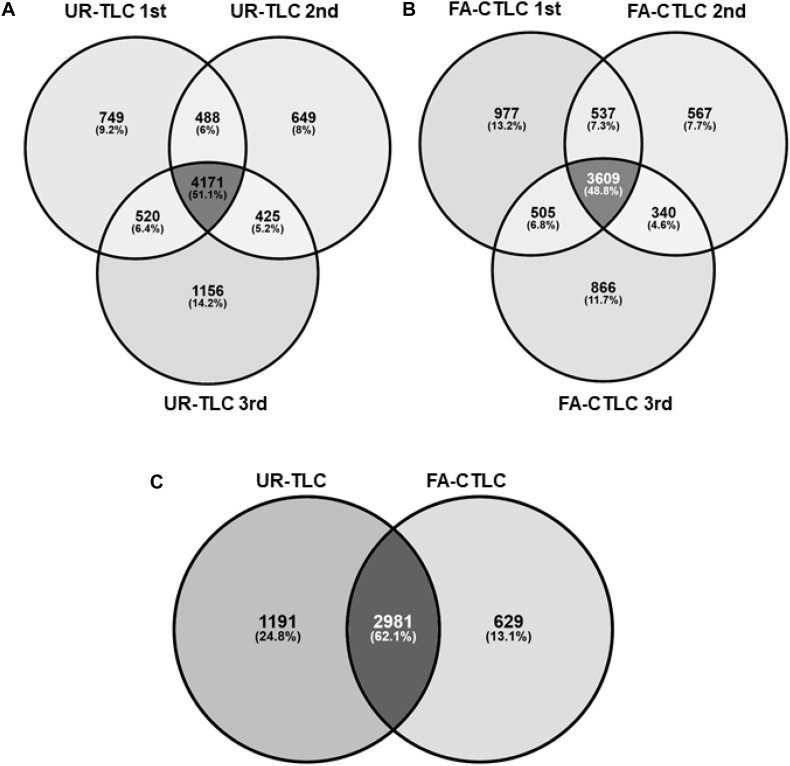
Analysis of the proteins identified by MS-MS after UR-TLC or FA-CTLC. **(A)** The reproducibility of protein identifications between the three biological repeats following UR-TLC treatment. **(B)** Reproducibility of protein identifications between the three biological repeats following FA-CTLC treatment. **(C)** A comparison of proteins identified by each treatment. The schematic diagrams were made by the Venny online tool (http://bioinfogp.cnb.csic.es/tools/venny/).

We assessed the reproducibility of the two methods by examining the proteins identified using three biological repeats from each treatment (Figure 2). When considering proteins with an FDR of <1%, we identified 4,171 protein groups common to all three biological repeats after UR-TLC and 3,609 protein groups common to all three biological repeats after FA-CTLC. The reproducibility of the proteins identified was 51.1 and 48.8% for the UR-TLC and FA-CTLC methods, respectively, (Figures 2A,B). We further compared the 4,171 protein groups common to all three biological repeats after UR-TLC (Figure 2A) to the 3,609 protein groups common to all three biological repeats after FA-CTLC (Figure 2B) and found that 2,981 groups of common proteins were identified by both methods (Figure 2C). Based on the results, 666 protein groups were unique to FA-CTLC identification method, while 1,523 protein groups were unique to UR-TLC. Supplementary Table 4 shows the complete list of protein groups identified in three biological repeats from both methods. The 1,523 UR-TLC-specific protein groups are shown in Supplementary Table 5 and the 666 FA-CTLC-specific protein groups identified after using the FA-CTLC are shown in Supplementary Table 6.

When considering proteins identified from three repeats, 7,946 protein groups were identified following UR-TLC. Protein groups (7,118) were identified following FA-CTLC (Supplementary Table 7). After combining the results derived from the two methods, we identified 8,993 protein groups in total (Supplementary Table 8). To determine the effectiveness of the two methods at identifying TMPs, the identified proteins were analyzed for the presence of TMDs and hydrophobicity.

### The FA-CTLC Method Preferentially Identifies TMPs With a Higher Number of TMDs

**FIGURE 3:**
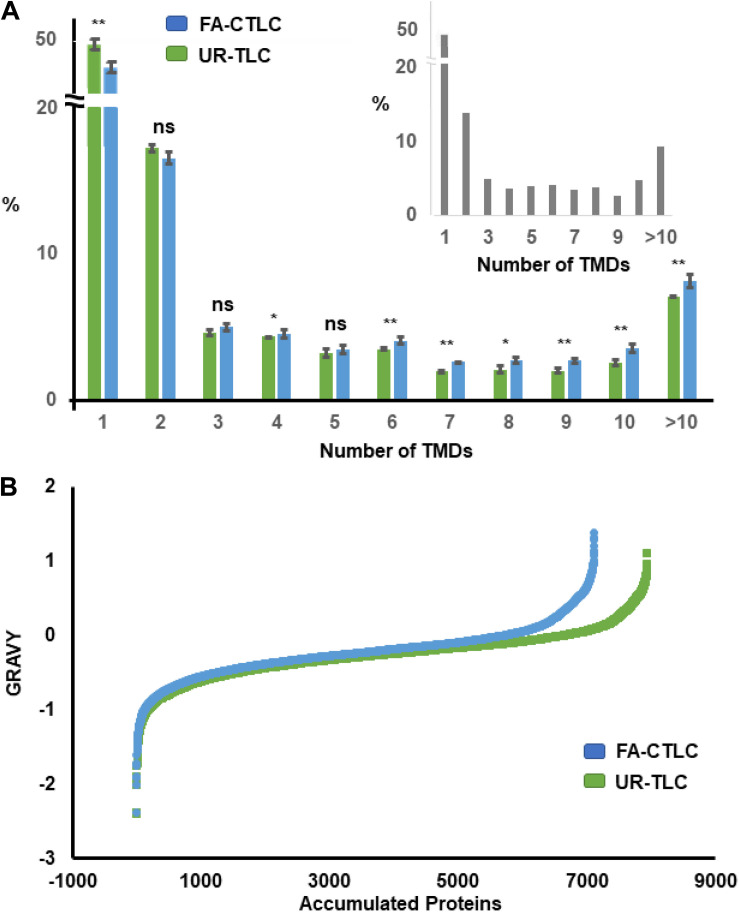
Distribution of proteins identified after applying the FA-CTLC or UR-TLC methods to *Medicago* microsomal membrane (MM) preparations from three biological repeats based on transmembrane domain (TMD) number and grand average of hydropathy (GRAVY) score. **(A)** The proteins identified after analyzing the UR-TLC- or FA-CTLC-treated samples from three biological repeats were submitted to the TMHMM server. The total predicted number of transmembrane protein (TMP) groups in the UR-TLC and FA-CTLC samples was 2,817 (35.45%) and 2,784 (39.11%), respectively. The predicted TMD distribution of the Medicago proteins in the UniProt database is shown in the inset panel as a comparison. There were 23.26% proteins predicted to be TMPs. *p ≤ 0.05, **p ≤ 0.01 (two-tail Student’s *t*–test). Error bars = standard error, *n* = 3. **(B)** The GRAVY scores were calculated (Kyte and Doolittle, 1982) from the proteins identified after analyzing the UR-TLC or FA-CTLC base on the previous published literature. Approximately 20% of proteins identified by FA-CTLC displayed a GRAVY score greater than zero and 17% of proteins identified by UR-TLC. Proteins with a hydrophobicity scores above 0 are more likely to be TMPs.

By using the TMHMM algorithm, 23.26% of the proteins in the *Medicago* Uniprot database were identified as TMPs. From the 7,946 protein groups identified after UR-TLC treatment, 2,817 protein groups (35.45%) contained at least one TMD, and of the 7,118 protein groups identified from the FA-CTLC treatment, 2,784 protein groups (39.11%) contained at least one TMD. Therefore, a higher percentage of TMPs can be identified by using the FA-CTLC method compared to using the UR-TLC method. Additional analysis showed that there were 5,129 protein groups identified from UR-TLC treatment and 4,334 protein groups identified from FA-CTLC treatment with 0 TMDs. This result suggests that the published procedures for removing non-membrane proteins (e.g., using sodium carbonate washes at pH 11) have poor efficacy. We further analyzed the TMP distribution in different biological repeats from each purification method using the TMHMM algorithm (Figure 3). About half of the TMD containing proteins identified using either method had only one TMD, and both purification methods gave no significant difference in distribution of TMDs to that predicted by analyzing the theoretical distribution of TMDs in all *Medicago* TMPs (inset of Figure 3, confirmed by Chi-Square Test, *p* = 0.32). This suggests that there is no major bias of either method in identifying TMPs and that the most abundant TMPs are likely to populate the lists of proteins identified. A significant difference between the proteins identified was that UR-TLC method preferentially identified proteins with only one TMD, whereas the FA-CTLC method preferentially identified more proteins with greater than four TMDs (Figure 3; *p* < 0.05). By combining the TMPs identified by UR-TLC and FA-CTLC, 3,289 TMP groups were identified, or 36.57% of all predicted TMPs.

The proteins identified after using UR-TLC or FA-CTLC methods were also examined using the GRAVY algorithm (Kyte and Doolittle, 1982). The GRAVY index indicates the hydrophobicity of the proteins, calculated by adding the hydropathy value for each residue and dividing by the length of the sequence. Proteins with a GRAVY scores above 0 are more likely to be hydrophobic proteins (Magdeldin et al., 2012). The GRAVY results (Figure 3B) showed that there were 1,420 (19.95%) protein groups identified by FA-CTLC and 1,313 (16.52%) proteins identified by UR-TLC, which displayed a GRAVY score greater than zero. These results indicated that the FA-CTLC method can preferably purify proteins that are hydrophobic.

**FIGURE 4:**
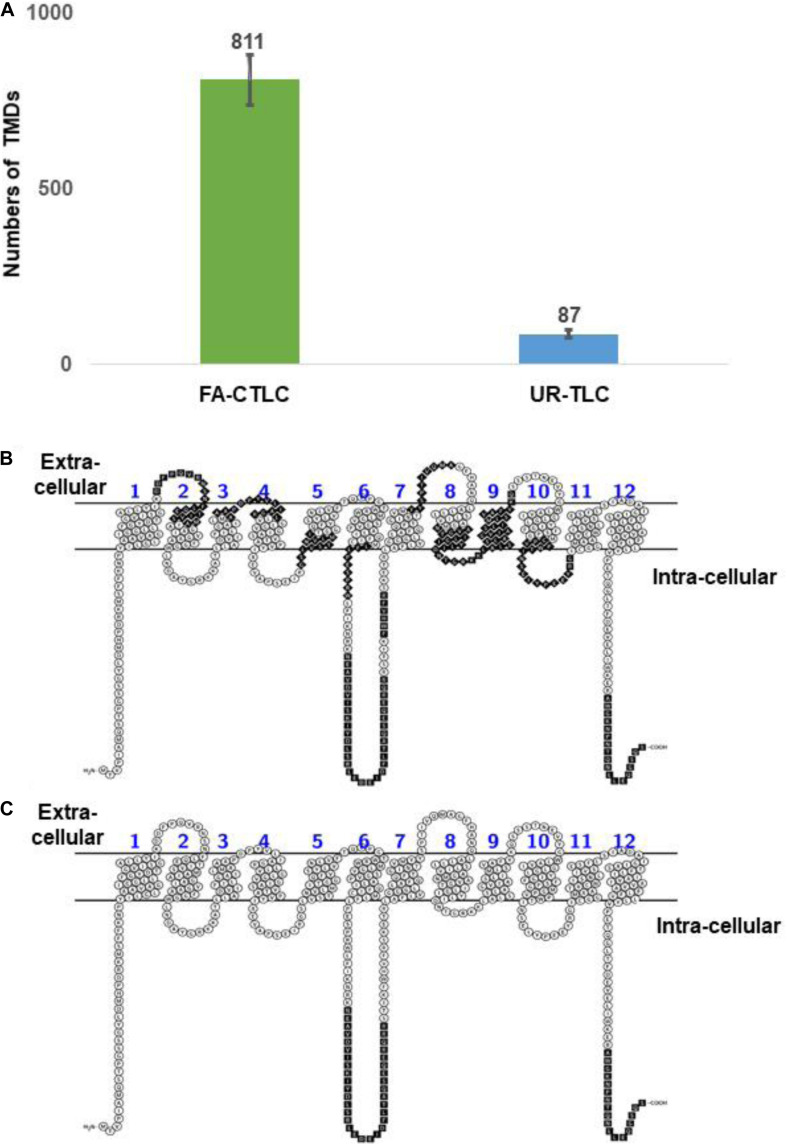
The FA-CTLC method preferentially identifies peptides with TMD motifs. **(A)** A comparison of the total TMDs identified numbers from both purification methods. The FA-CTLC methods identified 9.3-fold more TMDs than the UR-TLC method. **(B,C)** The peptides identified in the MTR_7g005910 transporter of *Medicago* after using the FA-CTLC **(B)** or UR-TLC method **(C)**. The peptides that were identified by MS were colored in black. The schematic diagrams were made using the Protter online tool (Omasits et al., 2014) (http://wlab.ethz.ch/protter/start/).

We developed the TRAMDOMI algorithm to identify the peptides that contain all or part of TMD motifs within the TMPs. This algorithm enabled us to quantify the relative ability of each method to identify peptides with TMD motifs. The search results showed that the FA-CTLC method can identify 9.36 times more TMD-containing peptides than the UR-TLC method (811 compared to 87; Figure 4A). Therefore, the results indicate that the FA-CTLC method is more effective at detecting peptides within TMPs that have TMD motifs, which boosts the number of TMPs identified. A list of identified TMPs and the TMDs identified using both purification methods is shown in Supplementary Table 9. To further illustrate the difference between the two methods, we compared the peptides identified for the MFS/sugar transporter (MTR_7g005910), which has 12 predicted TMDs (Figures 4B,C). Clearly, the FA-CTLC method identified more peptides within MTR_7g005910 with TMD motifs, whereas the UR-TLC method only identified MTR_7g005910 peptides predicted to loop into the cytoplasm, and none that contained TMD motifs.

### Transporter Proteins Are Preferentially Identified by Using the FA-CTLC Method

**FIGURE 5:**
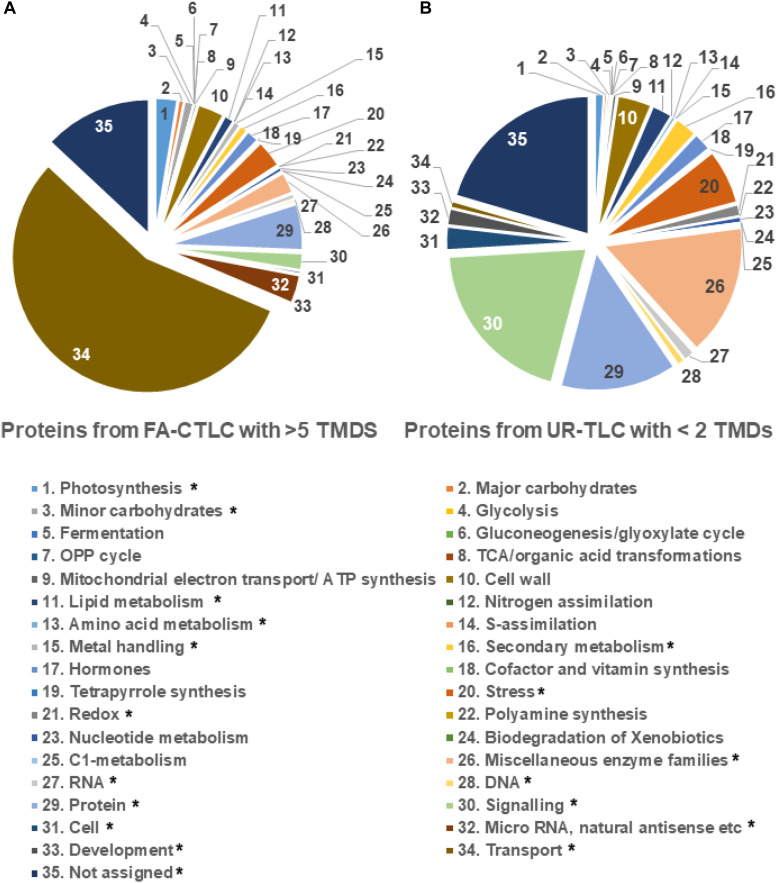
Functional analysis of the proteins preferentially identified using either the FA-CTLC or UR-TLC methods. **(A)** The proteins preferentially identified after using FA-CTLC (i.e., with >5 TMDs) were predominantly transporters. **(B)** The proteins preferentially identified after using UR-TLC (i.e., with one TMD) were predominantly signaling proteins. The category in which the FA-CTLC method had significant difference with *p*-value < 0.05 was labeled with *.

Given that each solubilization and cleavage method identified distinct classes of peptides, a Mercator analysis was done to determine if the two methods resulted in the enrichment of the identification of proteins with different functions. The results (Figure 5) show that the TMPs containing one TMD, which were preferentially identified by UR-TLC, were mostly functionally assigned as being signaling proteins (20.81%), and the proteins preferentially identified by FA-CTLC, which had four or greater TMDs were predominantly functionally assigned as being transporters (58.01%). We further examined the difference between two data sets by a binomial test. The results showed that the proteins identified by the FA-CLTC method were significantly different in 18 categories when compared to the UR-TLC method (Figure 5). The complete protein functional analysis list is shown in Supplementary Table 10, and the binomial test results are shown in Supplementary Table 11.

**FIGURE 6:**
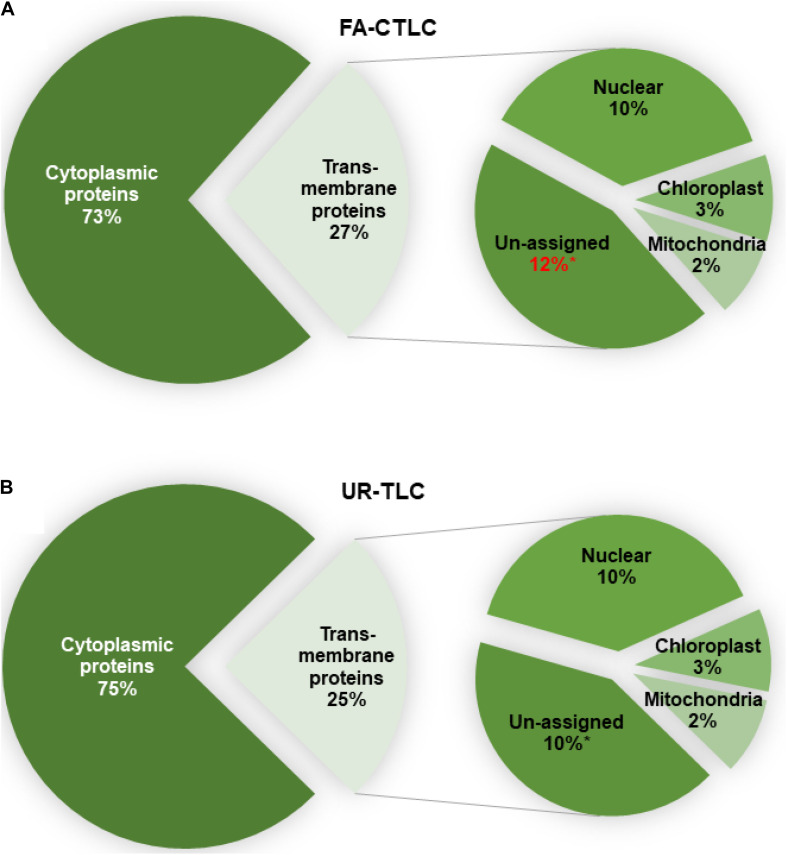
The predicted subcellular location of the proteins identified after using FA-CTLC or UR-TLC. **(A,B)** Between 73 and 75% of the proteins identified in the MM preparations were cytoplasmic proteins. Of the proteins identified to be TMPs, there was no significant difference in the identity of the proteins predicted to reside in the nuclear, chloroplast, or mitochondrial membranes. A *t*-test confirmed a significance difference (*p* < 0.05, *n* = 3) between the two methods in identifying proteins where the subcellular location could not be assigned (the “unassigned” category).

To determine the likely membrane where the TMPs identified reside, all proteins were analyzed for their subcellular location using LOCALIZER (Figure 6). Irrespective of the method used, an analysis of the TMPs identified showed that there was a similar distribution of proteins predicted to reside in the membranes of the nucleus, chloroplast, or mitochondria. For both methods, the FA-CTLC method identified significantly more TMPs where the subcellular location could not be assigned to an organelle (*p* = 0.044, *n* = 3). The complete subcellular location prediction list is shown in Supplementary Table 12.

## Discussion and Conclusion

The results showed that the FA-CTLC method was superior at solubilizing and digesting more hydrophobic proteins from MM preparations. Of the 57,065 proteins in the MT data base, 13,274 (23.26%) are predicted to be TMPs. The combined output of the two methods identified 3,289 TMP groups representing 36.57% of all TMPs, which is 1.5-fold more protein identifications achieved in a recent quantitative proteomic analysis of young Medicago seedlings (Long et al., 2018) and more comparable to the number of Medicago proteins identified using a similar sampling and bioinformatics procedure and similar instrumentation (Marx et al., 2016). In this study, we achieved a comparable number of identifications by a cost-effective method with less fractions and MS runs. About 50% of the TMPs identified using either method had only one TMD, but reassuringly, both purification methods gave no significant difference in the distribution of TMDs to that predicted by analyzing the theoretical distribution of TMDs in all Medicago TMPs using the THMMM algorithm. Therefore, this result suggests that there was no major bias of either method in identifying TMPs. To compare the TMD identification efficiency between the two methods utilized, we customized the TRAMDOMI algorithm to reveal how many TMDs were purified and identified from each purification method. The TRAMDOMI algorithm is the first python script designed for matching TMDs with peptides identified by MS. By using the TRAMDOMI algorithm, we identified a significant benefit of using the FA-CTLC method: this method preferentially identifies TMPs with a significantly higher number (9.4-fold) of TMDs than the UR-TLC method. In addition, each method identified partially non-overlapping TMP cohorts. Each purification method still had its preference, since 666 protein groups are unique to FA-CTLC identification method, while 1,523 protein groups are unique to UR-TLC. This result was validated by the FA-CTLC method identifying more transporter proteins, which have >8 TMDs, whereas the UR-TLC method preferentially enriched signaling proteins, which contain one TMD. The results implied that TMPs that were buried in cell membrane were difficult to denature or solubilize using 8 M urea. Therefore, UR-TLC method most likely shaves the exposed extra- and intracellular domains that loop away from the TMP regions imbedded inside the membrane. This deficiency leads to lower protein sequence coverage for proteins with a higher number of TMDs. By contrast, the UR-TLC method gave a better identification of TMPs with one TMD. TMPs from *Medicago* have a variable number of TMDs that range from 1 to over 30. Therefore, TMDs constitute a variable percentage of the composition of TMPs. TMDs are poorly represented in bottom–up MS (Kar et al., 2017), and the ability of a TMP to be detected by MS depends on its subcellular location, tissue specificity, natural abundance, the methodology used for fractionation, and the sensitivity and accuracy of the instrumentation (Bausch-Fluck et al., 2015; Itzhak et al., 2016; Reinke et al., 2017). Therefore, any MS-based method designed to improve the identification and coverage of TMPs should identify peptides from those parts of the TMPs that include the TMDs.

It is unclear if the acid-based solubilization or the preliminary cleavage at Met residues followed by the trypsin/LysC digestion is the basis for the improved TMD coverage in this work. Recently, Sun et al. (2020) used VAILase cleavage of purified proteins to marginally improve TMD coverage, although VAILase is not currently commercially available. Therefore, it is possible that using proteases such as VAILase, which cut at aliphatic amino acids (Val, Ala, Ile, Leu, and Thr), may improve TMD coverage (Sun et al., 2020).

Increasing the protein sequence coverage of TMPs is known to benefit quantitative proteomics (Ishihama et al., 2008; Millioni et al., 2011; Koziol et al., 2013). Therefore, since the FA-CTLC method can provide a higher sequence coverage of proteins with a higher number of TMDs, it has the potential to provide superior data for quantitative proteomics. We recommend that combining the two methods should achieve better TMP identification and a better coverage of TMP peptides. The reproducibility among the biological repeats could be further improved by employing label-free quantification (Mosley et al., 2011; Müller et al., 2018; Barkovits et al., 2020), which may further reveal the differences in TMP abundance between the two solubilization procedures. After combining the results derived from the two methods, we identified 8,993 protein groups and 3,289 TMPs in young shoot tissues. Therefore, if more tissues were examined and more extensive, membrane fractionation techniques applied, the number of TMPs and their coverage would be expected to increase.

## Data Availability Statement

The raw mass spectrometry data was submitted via ProteomeXchange (Vizcaíno et al., 2014) to the PRIDE repository (PXD022299) (Vizcaíno et al., 2012). TRANDOMI script is available on public server Github (https://github.com/HanChung-Lee/TRAMDOMI.git) for downloading.

## Author Contributions

HL and MD designed the research. HL performed the research. HL, ACa, AB, and MD wrote the manuscript. ACa wrote the python scripts and analyzed the data. BC and ACo performed the MS. All authors contributed to the article and approved the submitted version.

## Conflict of Interest

The authors declare that the research was conducted in the absence of any commercial or financial relationships that could be construed as a potential conflict of interest.

## Footnotes

1 http://www.cbs.dtu.dk/services/TMHMM/
